# Cognitive side-effects of electroconvulsive therapy: what are they, how to monitor them and what to tell patients

**DOI:** 10.1192/bjo.2020.17

**Published:** 2020-04-17

**Authors:** Richard J. Porter, Bernhard T. Baune, Grace Morris, Amber Hamilton, Darryl Bassett, Philip Boyce, Malcolm J. Hopwood, Roger Mulder, Gordon Parker, Ajeet B. Singh, Tim Outhred, Pritha Das, Gin S. Malhi

**Affiliations:** Treatment Algorithm Group; and Department of Psychological Medicine, University of Otago – Christchurch, New Zealand; Treatment Algorithm Group; and Department of Psychiatry, University of Melbourne, Australia; Treatment Algorithm Group; Academic Department of Psychiatry, Northern Sydney Local Health District; Sydney Medical School Northern, University of Sydney; and CADE Clinic, Royal North Shore Hospital, Northern Sydney Local Health District, Australia; Treatment Algorithm Group; and Private Practice in Psychiatry and Division of Psychiatry, the University of Western Australia, Australia; Treatment Algorithm Group; and Discipline of Psychiatry, Sydney Medical School, Westmead Clinical School, University of Sydney, Australia; Treatment Algorithm Group; School of Psychiatry, University of New South Wales; and Black Dog Institute, Australia; Treatment Algorithm Group; and School of Medicine, IMPACT Strategic Research Centre, Deakin University, Australia; Treatment Algorithm Group; Academic Department of Psychiatry, Northern Sydney Local Health District; Sydney Medical School Northern, University of Sydney; and CADE Clinic, Royal North Shore Hospital, Northern Sydney Local Health District, Australia

**Keywords:** Depression, cognition, electroconvulsive therapy, neuropsychological tests, memory disorders

## Abstract

**Background:**

Electroconvulsive therapy (ECT) is recommended in treatment guidelines as an efficacious therapy for treatment-resistant depression. However, it has been associated with loss of autobiographical memory and short-term reduction in new learning.

**Aims:**

To provide clinically useful guidelines to aid clinicians in informing patients regarding the cognitive side-effects of ECT and in monitoring these during a course of ECT, using complex data.

**Method:**

A Committee of clinical and academic experts from Australia and New Zealand met to the discuss the key issues pertaining to ECT and cognitive side-effects. Evidence regarding cognitive side-effects was reviewed, as was the limited evidence regarding how to monitor them. Both issues were supplemented by the clinical experience of the authors.

**Results:**

Meta-analyses suggest that new learning is impaired immediately following ECT but that group mean scores return at least to baseline by 14 days after ECT. Other cognitive functions are generally unaffected. However, the finding of a mean score that is not reduced from baseline cannot be taken to indicate that impairment, particularly of new learning, cannot occur in individuals, particularly those who are at greater risk. Therefore, monitoring is still important. Evidence suggests that ECT does cause deficits in autobiographical memory. The evidence for schedules of testing to monitor cognitive side-effects is currently limited. We therefore make practical recommendations based on clinical experience.

**Conclusions:**

Despite modern ECT techniques, cognitive side-effects remain an important issue, although their nature and degree remains to be clarified fully. In these circumstances it is useful for clinicians to have guidance regarding what to tell patients and how to monitor these side-effects clinically.

Despite considerable advances in the practice of electroconvulsive therapy (ECT), the benefits of this treatment remain a balance between effectiveness and the risk of cognitive side-effects.^1^ It is therefore important for clinicians not only to understand the clinical indications and likely clinical benefits of ECT, but also to have a clear understanding of the cognitive side-effects. However, the complexities of research evidence, different ECT techniques and differing cognitive tests and functions, make it increasingly difficult for busy clinicians to understand the latest evidence. Therefore, the aim of this paper is to assist clinicians by briefly discussing the evidence regarding the cognitive side-effects of ECT. We then consider whether screening of cognition during ECT can be developed in order to detect problems at an early stage and how this might be done. Finally, based on the current evidence, the paper outlines what information clinicians should present to patients regarding the potential cognitive side-effects of ECT. The issue of cognitive side-effects of ECT is illustrated using the example of treatment of major depressive episodes (MDEs), although similar issues probably apply to other diagnostic groups.

## Method

A Committee of clinical and academic experts from Australia and New Zealand met to the discuss the key issues pertaining to ECT and cognitive side-effects. Evidence regarding cognitive side-effects was reviewed, as was the limited evidence regarding how to monitor them. Both issues were supplemented by the clinical experience of the authors.

## Results

### What are the cognitive side-effects of ECT?

Several important factors determine the risk of cognitive side-effects of ECT. These can be divided into the following, all of which interact to determine risk.

- The domain of cognitive function that is being considered.
- ECT treatment parameters – electrode placement, dose of electricity, pulse width, treatment frequency, number of treatments.
- Individual patient factors.

### What domains of cognitive function are affected?

The meta-analyses of Semkovska and colleagues^2,3^ examined pooled data from existing studies. These divided functioning into separate cognitive domains and examined subacute (0–3 days), short-term (4 days–14 days) and long-term (14 days–2 years) effects. Though these meta-analyses have provided the most comprehensive overview to date, the results are complex and differences between the effects on different cognitive tasks may relate to properties of the tasks being employed rather than being a function of the sensitivity of that domain of cognitive function to disruption by ECT *per se*. It is also important to recognise that cognitive tasks inevitably rely on a range of functions and that it is difficult to separate these out. For example, memory tests inevitably also load on executive and attentional function whereas tests of attention load on executive function and vice versa. A simplified summary of the data is given below.

#### 

##### Non-memory cognition

In the subacute period following ECT (0–3 days), tests of executive function and processing speed were impaired compared with baseline. However, tests of attention were not impaired at this time point.^2^ When measured between 4 and 14 days most tests had improved significantly compared with baseline and none were significantly below baseline. The improvement is likely to have been a result of successful treatment of the depressive illness as studies suggest that in very severe depression, a high percentage of patients have very significant cognitive impairment.^4–6^

Longer term, with a wide range of follow-up intervals (2 weeks to 2 years), there was no evidence of impairment in tests of non-memory cognition.^2^ The only caveat to this may involve the use of sine wave ECT machines (discussed below).

##### Anterograde memory

Individual studies show that there was impairment on tests of anterograde memory from early in a course of ECT^7^ and meta-analyses suggest that this was still impaired subacutely (0–3 days) but returned to baseline (once again a baseline that may have been adversely affected by depression) between 4 and 14 days after the end of the course of ECT.^2^ The degree of initial impairment depends on treatment and patient factors considered below. Once again, the data refers to mean values and compares performance with a baseline, which is likely to be reduced secondary to the cognitive effects of severe depression itself.^5^

Beyond 14 days, there was no evidence of objectively assessed impairment.^2^

A caveat, which applies to all domains of cognitive function, is that although studies reporting the results of objective cognitive tests present mean results, the finding of no mean change from baseline does not necessarily mean that no patients experienced a clinically significant reduction in function. If, for instance, some patients improve greatly from baseline, a lack of mean change implies that some patients deteriorate. This is important to bear in mind when explaining risk on a case by case basis in clinical practice. Studies have rarely provided analysis on an individual level. We recommend that future studies provide this type of analysis in order for clinicians to be able to give information to patients regarding the percentage risk of significant decline.

##### Retrograde memory

When patients report cognitive side-effects it is often loss of autobiographical memory (i.e. loss of memory for events that the person has experienced) that are reported.^8^ However, both episodic memory (memory for experiences) and semantic memory (memory for facts) are affected. Retrograde amnesia for non-personal information has also been reported.^9^ Loss of memory in both areas may be distressing and functionally impairing. Objective testing of the loss of autobiographical memory (personal memories – including both episodic and semantic), usually using the Colombia University Autobiographical Memory Interview (CUAMI), has shown that the degree of loss differs depending on treatment parameters and that it exceeds the normal decay of memories over time in healthy matched participants. Furthermore, in studies where it is objectively measured for at least 1 year^10^ this loss has been found to persist. Whether it continues for longer than 1 year is not clear from objective studies, but some patients report that it does and it seems unlikely that lost memories could return spontaneously after this point. Although studies report loss of autobiographical memory as a mean, compared with baseline, identification of individuals who experience particularly severe loss has rarely been done. Where this has been done, there is evidence that females and those receiving bilateral ECT are more at risk.^11^

##### Subjective impairment

Studies of subjective reports of cognitive side-effects show varying percentages of patients who complain of ‘memory loss’, depending on a range of factors, including who interviewed them (Rose et al^8^). A recent review suggests that 60% of patients report memory problems, with 40% reporting that these lasted from several weeks to several years.^12^ However, most of the patients involved in these studies received bilateral ECT, limiting generalisation to other forms of ECT. Subjective assessment of cognitive problems following ECT, usually assessed using detailed questionnaires, such as the Cognitive Failures Questionnaire,^13^ correlates poorly with objective deficits. Subjective impairment often reduces throughout the course of treatment – tracking closely with clinical response. However, studies that used a simple question or seven-point Likert scale asking patients whether ECT ‘helped’ or ‘hurt’ their memory showed that scores on this scale declined (i.e. ECT ‘hurt’ memory on average) and this, in contrast with more complex assessment, correlated with loss of autobiographical memory.^14,15,16^

### How do treatment parameters affect cognitive side-effects?

Modern ECT can be broadly divided into three types depending on placement of electrodes and the pulse width of electricity administered.

- Bilateral ECT (bitemporal or bifrontal electrode placement) using a brief pulse width.
- Unilateral (usually right) (RUL) ECT using a brief pulse width – with pulses of electricity usually having a duration of 0.5–1 mS.
- Unilateral using an ultra-brief pulse width (UB-RUL), usually 0.25–0.3 mS.

Although overall efficacy increases from UB-RUL to RUL to bilateral,^17,18^ so do cognitive side-effects. In particular, reduction in scores on the CUAMI immediately following ECT follow the same gradient and are greater than for controls measured over the same time period. For each treatment, the dose of electricity above threshold also has an impact on the degree of cognitive side-effects (the higher the dose the greater the effects), including the long-term loss of autobiographical memory.^19^

#### Frequency of treatment

Few studies have examined the effects on cognitive side-effects of differences in frequency of treatment, in an RCT design. Although bilateral ECT three times a week provided more rapid relief, patients had more severe cognitive side-effects.^20^ In either case, effects are limited. A meta-analysis^2^ also showed some neurocognitive tests to be differentially affected by frequency when comparing results between studies (i.e. not randomised within studies), but only in the short term (4–14 days). This analysis did not consider retrograde amnesia.

#### Duration of treatment

ECT given at a frequency of two or three times a week causes increasing cognitive side-effects as the course lengthens.^7^ What has not been systematically studied is whether for most patients there is an acceleration of cognitive decline at a particular point in the course of treatment. An often recommended maximum number of treatments is 12 based on the fact that most patients who respond do so within that number of treatments. The mean number of treatments in most research studies and in clinical practice is between 8 and 10. Where UB-RUL is used the mean number of treatments may be slightly greater.^21^ Our clinical experience is that at some point later in a course of ECT (particularly bilateral) cognitive side-effects sometimes rapidly become more problematic (i.e. there is a threshold effect). Therefore, wherever clinically possible, prolonged courses (>12 treatments) should be avoided and cognitive effects need to be monitored closely if the course is extended.

#### Maintenance treatment

A related question is whether maintenance treatment causes progressive cognitive side-effects. Pertinent to this issue is, the frequency of maintenance treatment which, by convention and empirically, is often monthly, often reached by gradual tapering of frequency from an index course. Individual case reports have suggested that cognitive side-effects do not accumulate in such instances^22^ but the evidence is limited and there is no good evidence regarding the impact of treatment interval. Our clinical experience is that shorter intervals, particularly less than 3 weeks, can be problematic if treatment is prolonged. Monitoring of cognition may be particularly important if maintenance treatment at a shorter interval is unavoidable.

#### Sine wave ECT

Meta-analysis has shown that older machines that deliver ECT in the form of a sine wave give rise to significantly greater cognitive impairment than the brief or ultra-brief pulse machines that are now standard in most high-income countries.^2,11^ Of particular concern is data suggesting that ECT delivered using these machines resulted in impaired reaction time acutely and 6 months later.^11^ In some parts of the world these machines are all that is available and cannot be upgraded. However, their use may still be justified in certain clinical situations. In this case, being aware of and monitoring likely side-effects is particularly important.

### Individual patient factors

#### Individual risk factors for cognitive side-effects

Baseline Modified Mini-Mental State Examination (3MS)^23^ scores correlate with autobiographical memory loss, such that those with lower scores have greater loss post-ECT.^24^ In addition, although there is surprisingly little direct evidence, clinical experience suggests that patients who are elderly, have pre-existing brain injury or low intellectual ability are more vulnerable to the cognitive side-effects of ECT (see McClintock et al^25^ for a review). Regarding severe loss of autobiographical memory, there is some evidence that females are more vulnerable.^11^

Regarding medication concomitant with ECT, only lithium has been associated with significantly increased risk of cognitive side-effects with ECT (see Loo et al^26^ for a review). Cognitive side-effects and delirium should be monitored more closely in patients administered lithium, particularly at higher serum levels.^27,28^

#### Monitoring cognitive side-effects

It would appear almost axiomatic that since cognitive side-effects are distressing, and develop progressively over the course of treatment, that they should be monitored in some way. Indeed, as patients should be told that memory loss may occur and will often have learnt of this through media and the Internet, it is reassuring if this is addressed repeatedly throughout the course of ECT. Indeed, several practice guidelines have specifically recommended routine assessment of cognitive function during a course of ECT^29,30^ (see Rasmussen^31^ for a comprehensive review of recommendations). However, there are several problems with systematic cognitive monitoring.

- Testing patients at baseline with particularly severe depression and even psychotic or catatonic features is difficult and often simply not feasible.
- Repeat testing can be problematic as patients will remember tasks and may develop compensatory strategies over time.
- In many centres resources are limited and the regular use of a detailed cognitive test battery is impractical.

Further research is needed to determine optimal testing to monitor cognitive side-effects. Ideally it should be demonstrated that early changes in monitoring tests correlate with later changes and that a patient with a large reduction in function on a particular test after two to three sessions is likely to go on to develop even greater and possibly clinically significant and distressing impairment later in the course of ECT treatment. Furthermore, there is no research that clearly indicates at what level of reduction from baseline concern should be raised. However, even without clear research evidence at this point, there are several potential uses of monitoring cognitive side-effects.

#### Clinical planning

Regardless of the caveats above, some form of monitoring is clearly desirable as it has the potential to guide treatment decisions. As discussed previously, choice of treatment modality depends on balancing clinical response and the potential for cognitive side-effects and this balance needs to be reviewed at regular intervals throughout a course of treatment and used to inform all aspects of the course of treatment. Clinical examples of this are given in Appendix 1.

An aspect of clinical planning that is important but sometimes neglected is the planning of recommendations in the weeks immediately following ECT. At this point recovery of new learning and executive function is variable and deficits may have an impact on activities such as driving and work. Clinicians need to have some understanding of cognitive functioning at this point in order to advise on return to these sorts of activities.

#### Medicolegal issues

Patients who believe they have developed significant cognitive impairment following a course of ECT may take legal action. In this situation systematic cognitive testing carried out before and after ECT may be very useful in demonstrating the presence or absence of significant reduction in objectively measured cognitive function between pre-treatment and post-treatment and in demonstrating that the clinic is active in anticipating and dealing with this problem.

### How to monitor cognitive side-effects

Many clinics, even in high-income countries, will not have the resources to conduct an extensive cognitive battery and until such intensive monitoring has been shown to be useful clinically, although not discouraging this, we do not recommend it as routine. Therefore, testing must prioritise the most important issues and focus on these.

#### Candidate tests or domains for monitoring

##### Post-ictal disorientation

Patients may be disoriented for a variable period of time following an individual treatment. In the extreme case, this disorientation may persist in a fluctuating way for hours or days following a treatment – a post-ictal delirium. Sobin et al^24^ showed a correlation between time to orientation post-treatment and loss of autobiographical memory at 2 months after the end of the course. The correlation was driven by greater retrograde amnesia in a small group of patients who had recovery of orientation more than 30 min after the treatment. With most modern treatments it is unusual for this to happen, but recording time to re-orientation and reporting to clinicians when this is greater than, for example, 30 min is very simple and we recommend that this is done. More recently a small study confirmed the predictive effect of re-orientation across both RUL and bilateral ECT.^32^ A further and more rigorous development of this is the use of a specific questionnaire which, administered at 30 min post-ECT, in a small study correlated with loss of autobiographical memory at the end of the course.^33^ Staff and families monitoring patients between treatments should be aware of the symptoms of delirium.

##### Global cognitive scales

Many units use scales measuring global cognitive performance as a means of monitoring. The 3MS has been shown to be sensitive to differences short term (4–14 days) between 2 × weekly and 3 × weekly and between RUL and bilateral electrode placement, and is able to detect the detrimental effects of ECT in the subacute period (0–4 days).^2^ On this basis, it is potentially useful for monitoring. Other similar scales may also be used on the same basis if they are more familiar to clinicians or more available – for example the Montreal Cognitive Assessment^34^ or the Addenbrooke's Cognitive Examination.

##### Brief general cognitive batteries

Several previous papers have recommended shorter, more clinically practical batteries of cognitive tests that could be delivered at intervals throughout a course. Porter et al^35^ recommended a battery based on tests that had, in research studies, shown significant differences between different types of ECT (RUL versus bilateral) on the basis that these were therefore sensitive to aspects of ECT that could be modified if necessary. Data on the utility of this battery in clinical practice are awaited. An attempt has also been made to construct extremely short (few minutes) batteries of tasks, changes in which may be predictive of later cognitive difficulties.^36^ However, although correlations between changes in this brief cognitive battery and tests of anterograde memory post-ECT, there was no evidence that this was predictive of long-term loss of autobiographical memory. Standardised, computerised batteries of cognitive tests may be a method with future utility.

##### Anterograde amnesia

One of the most consistent findings, at least in the short term, is impairment of new learning, shown by reduced scores in verbal learning. This has often been demonstrated using the traditional Rey Verbal Learning Task^37^ or the shorter but less sensitive Hopkins Verbal Learning Test.^38^

##### Retrograde amnesia

The planned nature of ECT allows an assessment of stored memories, prior to the start of a course, in order to be able to monitor what has been lost. Usually, amnesia for autobiographic memory is measured. The most systematic approach to this is to elicit a large number of memories, record these and then prompt patients to retrieve the memories later in the course of treatment. This has been done most commonly and systematically using the CUAMI, a scale that was carefully constructed to illicit positive, negative and neutral memories and recent and remote events. The same questions are then asked at intervals throughout a course of ECT to generate a score reflecting the amount of information forgotten. This has been compared with the natural loss of memories over time in healthy participants.^10^ It is sensitive to differences in different methods of ECT, with the amount of information being forgotten at 6 months displaying a gradient from healthy participants at one end of the gradient to those having bilateral ECT at the other end, with unilateral ECT in between. The scale is, however, lengthy (281 questions over 1–3.5 h) making it impractical for use in clinical practice.

A shorter form was therefore developed (CUAMI- SF) that utilises only 30 questions, focusing on the previous 1 year. Areas covered are shown in Appendix 2 and illustrate the areas in which questioning has been able to elicit loss of memory. These items were chosen from the larger CUAMI because they produced high and equivalent rates of production of memories at baseline in patients with depression and controls, and because they differentiated RUL from bilateral ECT. Both the CUAMI and its short form have been criticised because they have not undergone the same rigorous standardisation as traditional neuropsychological tests.^39^ However, they represent a specific attempt to perform a particular function, which is to measure loss of autobiographical memory, in research into ECT and, in the case of the CUAMI-SF, to attempt to produce a scale that can be used clinically to measure autobiographical memory loss during and after ECT. To date, no better alternative has been developed.

Possible further criticisms may be that some of the items asked are poorly discriminative in certain people or populations with different customs or lifestyles and that the test may not therefore build up a picture of important memories that are likely to be lost. For example, in our practice, questions about what the patient did at New Year do not elicit a lot of variable information in the elderly people who have depression, who may easily be able to remember that they went to bed, as they do each year.

##### Subjective impairment

Although more complex questionnaires assessing subjective memory tend to correlate better with mood than objective impairment, simpler questions regarding whether ECT has ‘helped or hurt’ memory correlates well with objective measurement of memory impairment.^3,15^ Therefore, this simple question or a simple Likert scale may be very helpful in alerting clinicians to the onset of memory problems.

Individualising questions about autobiographical memory risks losing standardisation. This means that information regarding whether a patient has reached a threshold of memory loss that is likely to become clinically concerning is not available. However, where resources are scarce, a practical alternative to the CUAMI-SF is for clinicians to discuss with patients and their relatives what they have experienced in the previous 1 year and elicit and record some details that the clinician can attempt to elicit again at intervals during a course of ECT. This might involve relevant items from the CUAMI-SF and other items specific to the patient. Examples might include: have they attended a wedding? If so, record details of who was getting married, who attended, where it was held. Or have they attended a sporting event, and if so, who was playing. Suitable events could be suggested by family or friends.

Recent data suggests that although many patients have a subjective sense of memory problems following ECT, when patients perceptions of memory problems before the course of ECT are taken into account, a much lower percentage have a change in this.^40^ Therefore it is helpful to ask about and record perceived memory gaps both before and after treatment.

Family and friends are frequently asked about memory performance of patients receiving ECT but it is important to be specific about what is meant by ‘memory’ and what specific aspects are being enquired about.

### High-risk situations

Some situations may be particularly high risk and merit increased monitoring. Examples (but not an exhaustive list) include the following.

- Patients who are likely to be at high risk – for example, elderly patients, those who have had significant brain injury or some other source of vulnerability such as concomitant lithium prescription.
- When the course of treatment extends beyond 12 treatments. This may not apply to UB-RUL which is relatively cognitive sparing and more frequently requires a longer course for full remission.
- Maintenance ECT, particularly if this has been at a frequency greater than monthly or is long term (longer than 1 year). Monitoring could be done at relatively infrequent intervals (3 months to 1 year) making it more practical to undertake repeated testing.
- In low- and middle-income countries where it may not always be possible to conduct ECT with the most up to date equipment (see ‘Sine wave ECT’ above). In this situation monitoring which can be quickly undertaken by relatively unskilled personnel, according to the suggestions in Appendix 3, may be possible and guide treatment decisions.

## Discussion

### What should we tell patients?

The following list is a distillation of our understanding of the cognitive side-effects of ECT, which we believe incorporates the most important points that should be conveyed to patients and their families regarding this issue.

- ECT may cause loss of memory for things that patients have learnt or experienced. Some patients find this distressing but some do not.
- This memory loss is normally worse for the time immediately before ECT (3 months) – but sometimes extends back for up to 1 year and may occasionally extend beyond this.
- Usually this memory loss will improve significantly by 6 months but depression may result in residual problems and some of the memories for things that have been experienced will not return.
- Ability to learn new ‘things’ will be less for a short time after ECT – this is usually for a maximum of 2 weeks, at which point, this ability will be back to baseline. This may delay return to usual activities.
- Ability to plan things, concentrate and attend to things may be improved because the depression has been treated.
- Beyond possible effects of ECT on learning and memory, unfortunately severe depression itself is related to problems with learning and memory and for some people this does not improve completely even when the depression has considerably improved.

Each aspect of these side-effects should be emphasised more for patients whose ECT treatment parameters or risk factors make the problems more likely:

- bilateral (bitemporal/bifrontal) ECT;
- prolonged course of standard treatment or frequent maintenance treatment;
- existing cognitive difficulties (brain injury, intellectual disability, other existing brain disease);
- elderly patients.

It is noteworthy that although evidence suggests a return to baseline and even improvement within 14 days of the end of a course of ECT, patients may be attempting to return to their roles in this period, something that could be hampered even by a brief period of short-term cognitive dysfunction. Patients should be warned about this possibility and clinicians should attempt to gauge the extent of the problem. Even temporary impairment may set up a cycle of difficulty in functioning, negative appraisal of performance and avoidance of cognitive activity, which in turn may contribute to suboptimal recovery or to relapse.

It is important to provide written information including to family/support people as patients may not remember explanations later. Most professional bodies have written information and health authorities usually have information sheets provided with consent sheets. These do not necessarily replace informed discussion.

### Implications

ECT is a potentially life-saving treatment for severe MDEs and it is frequently effective in cases of treatment-resistant MDEs. The main disadvantage is that it is associated with cognitive side-effects. Some forms of ECT minimise the risk of these side-effects but in some patients this is at the expense of reduced efficacy. Despite considerable advances in technique there is still the need to strike a careful balance between efficacy and side-effects. The most important area of cognitive effects associated with ECT is loss of autobiographical memory, which is distressing for some patients, and all patients should be informed of the possible nature and extent of this phenomenon. Data suggesting a lack of effect on other aspects of cognition is based on group means and does not exclude the possibility that a subgroup of patients experience deficits that are masked by a general improvement in cognition in the rest of the group.

Monitoring is demanding and research findings regarding the best way of monitoring is limited, but some basic monitoring can be achieved relatively easily. It is important to give very clear information to patients and their families.

## Data Availability

Data sharing is not applicable to this article as no new data were created or analysed in this study.

## Appendix 1

### Clinical examples of monitoring in practice

#### Example one

A 78-year-old with moderate depression and treatment resistance shows minimal response after six treatments with ultra-brief right unilateral (RUL) electroconvulsive therapy (ECT) despite increases in dose. Cognitive monitoring suggests no deterioration. Therefore, a decision is made to change to brief pulse RUL.

#### Example two

A 40-year-old with severe, suicidal and psychotic depression shows significant improvement but still presents with moderate symptoms after six treatments with bilateral ECT. Cognitive monitoring shows a very significant loss of autobiographical memory. In this situation the decision was made to switch to RUL ECT until full remission was achieved.

#### Example three

A 25-year-old with a history of a moderate traumatic brain injury and current severe depression with suicidal thoughts is treated with RUL ECT. There is some improvement but very significant loss of autobiographical memory. The decision was made to switch to ultra-brief RUL ECT to achieve further improvement in mood symptoms.

## Appendix 2

Features of the Columbia University Autobiographical Memory Interview – Short Form:

- Focuses on past 1 year.
- Asks about work history, travel, relatives, celebrations (birthday and New Year), and medical treatment.
- Probes each area with five further questions – for example, travel (number of days away, name of lodging, travel companions, reason for trip and what was enjoyed about the trip).
- Subsequent scores based on information given at baseline and can be expressed as percentage lost.
- Takes 10–20 min to administer.

## Appendix 3

### Recommendations for monitoring of cognitive function during and after electroconvulsive therapy

#### Minimal screening

- Record and report any failure to be oriented more than 30 min after a treatment.
- Educate all carers regarding features of inter-ictal delirium.
- Enquire at baseline about important events in the patient's life during the previous 1 year. Elicit and record some salient details. Discuss with family/support people and confirm. Ask about these events regularly (every three treatments). Record whether there is a significant loss of memory for these events.
- Enquire of patient and relatives (and nursing staff if the patient is an in-patient) regarding any evidence of memory loss both prior to and after treatment. Explain to all three groups what autobiographical memory is and the types of memory that may therefore be lost.
- Ask patients whether they believe the treatment has ‘helped’ or ‘hurt’ their memory and consider rating on a scale of one to seven, with four being neutral.

#### If resources exist

- Consider doing the Modified Mini-Mental State Examination or similar brief cognitive screen
- Consider undertaking a formal Columbia University Autobiographical Interview – Short Form.
- Consider doing a test of anterograde memory for example (Hopkins Verbal Learning Test^30^ or Rey Verbal Learning Test^29^).

#### Frequency

- Baseline and after every two to three treatments.
- More frequently in high-risk situations.
- Three to four days after the last treatment of the course of electroconvulsive therapy to guide planning of return to activities.
